# Alterations in Adenosine Metabolism and Signaling in Patients with Chronic Obstructive Pulmonary Disease and Idiopathic Pulmonary Fibrosis

**DOI:** 10.1371/journal.pone.0009224

**Published:** 2010-02-16

**Authors:** Yang Zhou, Jayasimha N. Murthy, Dewan Zeng, Luiz Belardinelli, Michael R. Blackburn

**Affiliations:** 1 Department of Biochemistry and Molecular Biology, University of Texas-Houston Medical School, Houston, Texas, United States of America; 2 Division of Pulmonary, Critical Care, and Sleep Medicine, University of Texas-Houston Medical School, Houston, Texas, United States of America; 3 Graduate School of Biomedical Sciences, University of Texas Health Science Center, Houston, Texas, United States of America; 4 Gilead Sciences, Palo Alto, California, United States of America; Emory University, United States of America

## Abstract

**Background:**

Adenosine is generated in response to cellular stress and damage and is elevated in the lungs of patients with chronic lung disease. Adenosine signaling through its cell surface receptors serves as an amplifier of chronic lung disorders, suggesting adenosine-based therapeutics may be beneficial in the treatment of lung diseases such as chronic obstructive pulmonary disease (COPD) and idiopathic pulmonary fibrosis (IPF). Previous studies in mouse models of chronic lung disease demonstrate that the key components of adenosine metabolism and signaling are altered. Changes include an up-regulation of CD73, the major enzyme of adenosine production and down-regulation of adenosine deaminase (ADA), the major enzyme for adenosine metabolism. In addition, adenosine receptors are elevated.

**Methodology/Principal Findings:**

The focus of this study was to utilize tissues from patients with COPD or IPF to examine whether changes in purinergic metabolism and signaling occur in human disease. Results demonstrate that the levels of CD73 and A_2B_R are elevated in surgical lung biopsies from severe COPD and IPF patients. Immunolocalization assays revealed abundant expression of CD73 and the A_2B_R in alternatively activated macrophages in both COPD and IPF samples. In addition, mediators that are regulated by the A_2B_R, such as IL-6, IL-8 and osteopontin were elevated in these samples and activation of the A_2B_R on cells isolated from the airways of COPD and IPF patients was shown to directly induce the production of these mediators.

**Conclusions/Significance:**

These findings suggest that components of adenosine metabolism and signaling are altered in a manner that promotes adenosine production and signaling in the lungs of patients with COPD and IPF, and provide proof of concept information that these disorders may benefit from adenosine-based therapeutics. Furthermore, this study provides the first evidence that A_2B_R signaling can promote the production of inflammatory and fibrotic mediators in patients with these disorders.

## Introduction

Destructive lung disorders such as chronic obstructive pulmonary disease (COPD) and interstitial lung disease such as idiopathic pulmonary fibrosis (IPF) affect millions of individuals and result in billions of dollars in annual health care cost. Considerable information has been gathered concerning the mechanisms that promote inflammatory and tissue remodeling processes in these disorders; however, relatively little is known about the pathways that drive their progressive and chronic nature. Deregulated or overactive wound healing pathways are hypothesized to contribute to the excessive remodeling responses that are seen in chronic lung diseases [1], [2]. Extracellular adenosine is a signaling molecule that is produced in response to cell damage and can regulate tissue injury and repair [3]. Consistent with this, adenosine levels are elevated in the lungs of patients with chronic lung disease [4], [5], [6], where it is hypothesized to regulate the balance between tissue repair and excessive airway remodeling [7]. Adenosine influences cell function by engaging G-protein coupled adenosine receptors that access a variety of intracellular signaling pathways [8]. Four adenosine receptors have been described: A_1_R, A_2A_R, A_2B_R and A_3_R. These receptors may have different affinities for adenosine and different cellular and tissue distribution. Levels of adenosine receptors are altered in the lungs of asthmatics [9], [10] and COPD patients [10], [11] and a recent study has shown that the A_2B_R is increased in remodeled airway epithelial cells of rapidly progressing IPF patients [12].

It has been recognized that adenosine may also play a critical role in the pathogenesis of chronic inflammatory disorders of the airways such as asthma and COPD.[13], [14]. For example, exogenous adenosine can elicit acute bronchoconstriction in patients with asthma or COPD [15], [16], while having no effect on normal individuals, suggesting a fundamental difference with regard to adenosine signaling in these patients. Activation of adenosine receptors can also influence the activity of cell types that play a central role in chronic lung disease including mast cells [17], eosinophils [10], macrophages [18], airway epithelial cells [19], pulmonary fibroblasts [20], and airway smooth muscle cells [21]. Moreover, recent studies directly demonstrate the role of adenosine in the regulation of pulmonary fibrosis. Exposure of human pulmonary fibroblasts to adenosine promotes their differentiation into myofibroblasts through activation of the A_2B_R [20]. In addition, activation of A_2B_R promotes the production of fibronectin from type II alveolar epithelial cells [22], a process that can impact pulmonary fibrosis. Collectively, these studies demonstrate that adenosine can regulate processes that influence pulmonary fibrosis and implicate the A_2B_R as a pro-fibrotic receptor.

**Figure 1:**
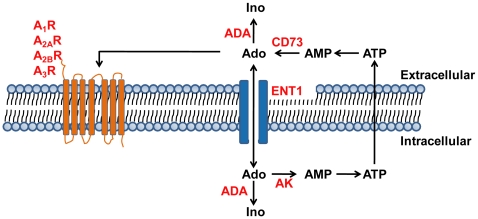
Key components of adenosine metabolism and signaling. In response to cellular stress and damage, ATP is released into the extracellular space and is rapidly dephosphorylated by extracelluar nucleotidases. CD73 catalyzes the formation of extracellular adenosine from AMP. Extracellular adenosine can interact with seven-transmembrane adenosine receptors, A_1_R, A_2A_R, A_2B_R, and A_3_R, which are coupled by heterotrimeric G proteins to various second messenger systems, or it can be transported into cells via facilitated nucleoside transporters, such as ENT1. Both extracellular and intracellular adenosine can be deaminated to inosine by adenosine deaminase (ADA). Intracellular adenosine can be secreted or phosphorylated back to ATP. The first step in this process is catalyzed by adenosine kinase (AK).

Examination of adenosine levels in animal models of chronic lung disease corroborate with these findings in humans. Transgenic mice that over express the Th2 cytokines IL-4 or IL-13 in the lungs develop progressive pulmonary inflammation and injury characterized by eosinophilic and monocytic infiltrates, fibrosis and alveolar airspace destruction in association with increases of adenosine in the lungs [23], [24]. In addition, adenosine levels are elevated in the lungs of mice exposed to the fibrosis inducing agent bleomycin [25] or following chronic ovalbumin exposure [26]. Lastly, there are correlations between the degree of inflammation and damage and adenosine accumulations in adenosine deaminase (ADA)-deficient mice [27]. In these animal models, levels of key components of adenosine metabolism (Figure 1) and signaling are altered. These changes include the up-regulation of ecto-5′-nucleotidase (CD73) [25], one of the key enzymes of adenosine production, and the down-regulation of ADA, one of the key enzymes for adenosine metabolism [23], [24]. In addition, we have observed up-regulation of pro-inflammatory and pro-fibrotic adenosine receptors in these models [23], [24], [25], [27], [28]. Moreover, therapeutic strategies to lower adenosine levels or inhibit adenosine receptors lead to improvement in pulmonary pathologies in many of these animal models [23], [24], [27], [29], [30], [31]. These findings suggest that adenosine-based therapeutics may be beneficial in the treatment of chronic lung diseases such as COPD and IPF.

The objective of the current study was to determine whether adenosine metabolism and signaling are altered in patients with COPD and/or IPF. Our hypothesis was that purinergic metabolism and signaling components are altered in a manner that promotes adenosine production in tissue samples from patients with COPD and IPF. Our results demonstrate that CD73 and the A_2B_R are elevated in lung biopsy samples from patients with Stage 4 COPD and Severe IPF compared to patients with preserved lung function. Expression of CD73 and the A_2B_R were localized predominantly to alternatively activated macrophages in airspaces. These elevations were associated with significant alterations in the expression of pro-inflammatory mediators known to be driven by A_2B_R signaling, and ex vivo studies demonstrated that activation of A_2B_R can influence the production of key inflammatory and fibrotic mediators from macrophages isolated from these patients.

## Methods

### Subjects

**Figure 2:**
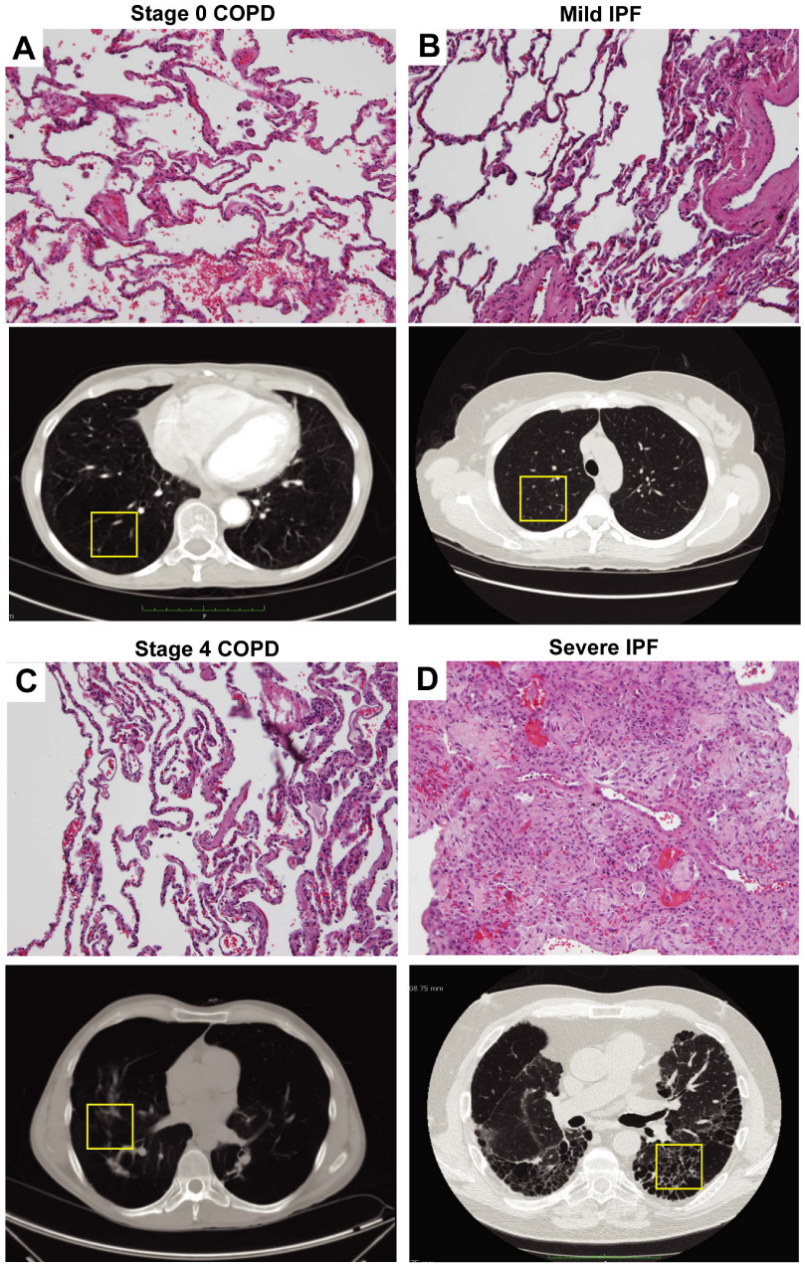
Lung histopathology and high-resolution CT scans. Representative H&E staining and hign-resolution CT scan images showing: (A) A Stage 0 COPD patient with preserved lung function. (B) A Mild IPF patient with preserved lung function. (C) A Stage 4 COPD patient. (D) A Severe IPF patient. Sections are representative of 10–14 different patients from each group. The yellow boxes in the CT scan images represent the approximate location of the surgical biopsies for obtaining tissues sections frozen material for analysis in these examples.

The use of human material for this study was reviewed by the University of Texas Health Science Center at Houston Committee for the Protection of Human Subjects. All studies in tissues were from existing samples already collected and deidentified and were therefore considered exempt. The analysis of lavage isolated from patients was also reviewed and approved by this committee with no ethical concerns. Surgical lung biopsy tissue samples were obtained from the Lung Tissue Research Consortium (LTRC) (Table 1). Patients were classified as Stage 0 COPD, Stage 4 COPD, Mild IPF and Severe IPF according to spirometry, pathological examination and high resolution CT scan (Figure 2). Stage 0 COPD and Mild IPF patients with preserved lung functions were used as controls in comparison to Stage 4 COPD and Severe IPF patients. For ex vivo culture experiments, macrophages were isolated form lavage fluid collected from Stage 4 COPD or Severe IPF patients that were lavaged as part of routine diagnostic procedures.

**Table 1:** Study population.

Parameter	Stage 0 COPD^1^	Mild IPF^2^	Stage 4 COPD^3^	Severe IPF^4^
N	4	10	10	10
Age, yrs	69 (61–78)	60 (50–77)	50 (44–63)	54 (26–62)
Sex, M/F	1/3	6/4	6/4	7/3
Pack-yrs smoking	25 (25–50)^a^	8 (3–10)^b^	36 (1–120)^c^	20 (3–32)^d^
Smoking status				
Ever/Never	4/0	5/4^e^	10/0	4/5^e^
FEV1, % pred	86 (84–89)	92 (66–109)	20 (12–40)*	38 (30–46)^#^
FVC, % pred	107 (80–113)	89 (80–105)	54 (13–77)*	38 (25–43)^#^
FEV1/FVC, %	60 (50–80)	80 (60–90)	30 (20–60)*	90 (70–100)

Data are presented as median (interquartile range). M/F: male/female; FEV1: forced expiratory volume in one second; % pred: % predicted; FVC: forced vital capacity. ^1^: Stage 0 COPD is defined as FEV1, % pred >80; ^2^: Mild IPF is defined as FVC, % pred >80; ^3^: Stage 4 COPD is defined as FEV1, % pred <50; ^4^: Severe IPF is defined as FVC, % pred <50; ^a^: data available for 3/4 Stage 0 COPD patients; ^b^: data available for 5/10 Mild IPF patients;^ c^: data available for 9/10 Stage 4 COPD patients;^ d^: data available for 4/10 Severe IPF patients;^ e^: data available for 9/10 Mild or Severe IPF patients. *: p<0.05 compared with Stage 0 COPD patients; ^#^: p<0.05 compared with Mild IPF patients.

### Quantitative RT-PCR

Total RNA was isolated from frozen lung tissue using Trizol reagent (Invitrogen Corp.). RNA was purified through an RNA-purification column (Qiagen) and treated using RNase-free DNase (Invitrogen Corp.). Transcript levels were quantified using Taqman real-time quantitative RT-PCR. Primer sequences for the transcripts examined are found in Table 2. Specific transcript levels were determined through comparison to a standard curve generated from the PCR amplification of template dilutions, and normalized to 18S ribosomal RNA and presented as mean transcript levels expressed as %18S RNA.

**Table 2:** Primer pairs and internal probe sequences.

Gene	Accession Number	Sequences
CD73	NM_002526	1447+GACAGAGTAGTCAAATTAGATG 1511−TGAGAGGGTCATAACTGG 1471+FAM TCTTTGCACCAAGTGTCGAGTGC
ADA	NM_000022	264+CTGCTGAACGTCATTGG 340−GCAGGCATGTAGTAGTC 281+FAM CATGGACAAGCCGCTCACCC
AK	NM_006721	1092+CCACTATGCAGCAAGCATC 1156−GGAAGTCTGGCTTCTCAGG 112+FAM TAATTAGACGGACTGGCTGCACCTT
ENT1	NM_001078177	1413+CCAGCCGTGACTGTTGAG 1489−CAGGACACAGGAATGAAGTAAC 1438+FAM CCAGCATCGCAGGCAGCAGC
A_1_R	NM_000647	1147+GCTGGCTGCCTTTGCAC 1215−GGATGCTGGGCTTGTGG 1165+FAM TCCTCAACTGCATCACCCTCTTCTGC
A_2A_R	NM_000675	838+ATGCTGGGTGTCTATTTGCG 902−TGGCTCTCCATCTGCTTCAG 865+FAM CTGGCGGCGCGACGACA
A_2B_R	NM_000676	977+CACTGAGCTGATGGACCACTC 1040−CAGTGACTTGGCTGCATGG 1018−FAM TCCCGCTGGAGGGTGGTCCT
A_3_R	NM_000677	708+CCCTACAGACGGATCTTGCTG 777−TGTTGGGCATCTTGCCTTC 734+FAM CCTGTCCCTGTGGAGGTTCCCCT
IL-6	NM_000600	153+ CCCCCAGGAGAAGATTCCA 223− TCAATTCGTTCTGAAGAGGTGAG 173+FAM 223− TCAATTCGTTCTGAAGAGGTGA
IL-8	NM_000584	100+TCTTGGCAGCCTTCCTGA 182−GCACTGACATCTAAGTTCTTTAGCACT 121+FAM CTGCAGCTCTGTGTGAAGGT
OPN	NM_000582	619+GGACTGAGGTCAAAATCTAAGAAG 693−GGTGATGTCCTCGTCTGTAG 646+FAM CGCAGACCTGACATCCAGTACCCT

### Enzyme Activity Assay

Lungs were homogenized and lysed on ice with protein lysis buffer (50mM Tris pH7.4, 150mM NaCl, 1% Triton-X 100, 0.1% SDS, 0.5% Na deoxycholate) freshly supplemented with 1X protease inhibitor cocktail (Roche Diagnostics). Lysates were vigorously vortexed and cleared by centrifugation at 14,000rpm for 15 min at 4°C. To quantify CD73 enzyme activity, 5 µg of protein extracts were incubated with 100 µM AMP at 37°C for 30 min in the presence of 1 µM deoxyconformycin in HEPES buffer, with or without 100 µM CD73 inhibitor adenosine- 5′- O- (α, β- methylenediphosphate) (AOPCP). To quantify ADA enzyme activity, 10 of µg protein extracts were incubated with 0.2 mM adenosine at 37°C for 60 min in HEPES buffer with or without 1 µM deoxyconformycin, an ADA inhibitor. Heat-inactive protein extracts were used as negative controls. Reactions were terminated at 95°C for 5 min. Reaction mixtures were then analyzed by reversed-phase (C18) HPLC, which permitted direct separation, identification, and quantification of enzymatic products [27]. Enzyme specific activity is given as nmol product formed per min per mg protein (nmole/min/mg protein).

### Histology and Immunostaining

HOPE or paraformaldyhyde fixed lung samples from the same location as RNA and protein lysates were dehydrated, and embedded in paraffin, and sections (5 µm) were collected on microscope slides and stained with H&E (Shandon-Lipshaw) according to manufacturer’s instructions. For CD73 immunostaining, HOPE-fixed sections were deparaffinized in isopropanol at 60°C and rehydrated in 70% acetone. Rehydrated slides were quenched with 1% hydrogen peroxide and endogenous avidin and biotin blocked with a Biotin Blocking System (DAKO Corp.). Slides were incubated with mouse anti-human CD73 antibody (Hycult Biotechnology, 1∶50 dilution, overnight at 4°C). For A_2B_R immunostaining, rehydrated slides were quenched with 1% hydrogen peroxide, antigen retrieval performed (Dako Corp.), and endogenous avidin and biotin blocked with a Biotin Blocking System (DAKO Corp.). Slides were incubated with rabbit anti-human A_2B_R Antibody (Chemicon, 1∶500 dilution, 1 hr at room temperature). Sections were incubated with ABC Streptavidin reagents and appropriate secondary antibodies, then developed with 3, 3′-diaminobenzidine (Sigma-Aldrich) and counterstained with methyl green. The number of positively stained inflammatory cells in each group was performed by counting positive cells in 20 images of each lung section at 10X magnification using Image Pro Plus software (Cybernetics). For immunofluorescence on tissue sections, rehydrated slides were fixed in 1∶1 acetone-methanol and treated with 1% NaBH_4_. Slides were blocked in 1% BSA, and incubated overnight at 4°C with the primary antibodies. For immunofluorescence on primary human macrophages, cells were cytospun and fixed in 3.7% paraformaldehyde in PBS and permeabilized in cold Methanol. Slides were blocked with 1% rabbit serum and incubated overnight at 4°C with the primary antibodies. Primary antibodies include: mouse anti-human CD73 antibody (Hycult Biotechnology, 1∶50 dilution), Rabbit Anti-human A_2B_R Antibody (Chemicon, 1∶500 dilution), rat anti-human MMR (CD206) antibody (R&D Systems, 1∶50 dilution). Sections and cells were incubated with the following secondary antibodies: Alexa Fluor 488 rabbit anti-mouse IgG, Alexa Fluor 488 goat anti-rabbit IgG, Alexa Fluor 568 goat anti-rat IgG (Intritrogen) then coversliped with Vectashield with DAPI (Vector Laboratories).

### *In vitro* Stimulation of Human Primary Alveolar Macrophages

Primary alveolar macrophages were obtained from BAL fluid of stage 4 COPD or severe IPF patients. BAL fluid was spun and cell pellets were resuspended in RPMI1640 containing 10% FBS and 10,000 U/ml penicillin/streptomycin. Cells were portioned into aliquots of 2×10^5^ cells/well, allowed to adhere for 4 hours at 37°C 5% CO_2_, and then rinsed twice with RPMI1640 without FBS. Cells were either pre-incubated with 100 nM CVT-6883 (selective A_2B_R antagonist) for 30 min followed by NECA or incubated with NECA alone (in DMSO, 10 µM NECA/well; Tocris Bioscience) for 12 h at 37°C 5% CO_2_. Tissue culture supernatants were collected and IL-8 and IL-6 levels were quantified using Human Quantikine ELISA kits (R&D Systems).

### Statistics

Groups were compared by analysis of variance; follow-up comparisons between groups were conducted using 2-tailed Student's t test. Associations between transcript levels of two genes were established by linear regression. Correlation significances were analyzed using Pearson correlation calculator software. Values are expressed as mean ± SEM. A p value of ≤0.05 was considered to be significant.

## Results

Transcript Levels of Components of Adenosine Metabolism and Signaling are Altered in the Lungs of COPD and IPF Patients

**Figure 3:**
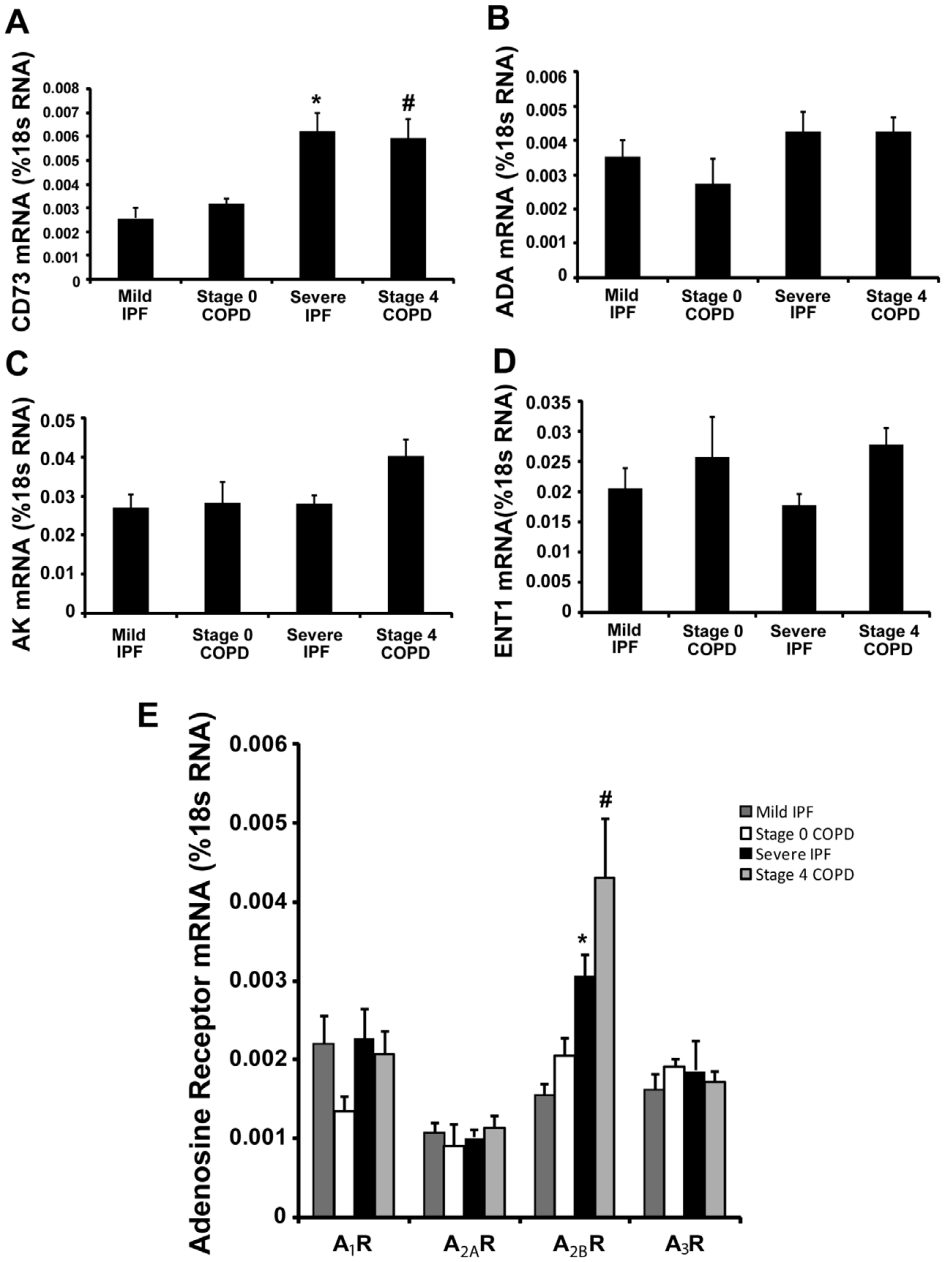
Expression of components of adenosine metabolism and signaling. Transcript levels of various enzymes in adenosine metabolism, and adenosine receptors were quantified in lung RNA extracts from patients using quantitative RT-PCR. Shown are levels of (A) CD73, (B) ADA, (C) AK, (D) ENT1, (E) Adenosine receptors. Results are presented as mean percentage of 18sRNA transcripts ± SEM. *p≤0.05 versus Stage 0 COPD. #p≤0.05 versus Mild IPF. n = 4 (Stage 0 COPD), n = 10 (Mild IPF), n = 8 (Stage 4 COPD and Severe IPF).

Components of adenosine metabolism and signaling are altered in mouse models of chronic lung disease in association with elevated levels of adenosine [23], [24], [25], [27] (Figure 1). Total RNA was isolated from surgical lung biopsy specimens and real-time RT-PCR was performed to quantify key components of adenosine metabolism and signaling. Results demonstrated a 3 fold increase of CD73 transcripts in RNA extracts from Severe IPF and Stage 4 COPD patients compared to Mild IPF and Stage 0 COPD patients with preserved lung function (Figure 3A). However, the transcript levels of ADA, AK and ENT1 were not altered (Figure 3B-D). All four adenosine receptors were detectable in subjects with preserved lung function (Figure 3E); however, only transcript levels for the A_2B_R were significantly increased in Severe IPF and Stage 4 COPD patients (Figure 3E). Interestingly, smoking status had no influence on the level of adenosine receptor expression in the IPF patients examined (data not shown). Collectively, these findings demonstrate elevations in components of adenosine production and signaling in the lungs of patients with progressive chronic lung diseases.

### CD73 and ADA Enzymatic Activities Are Altered in the Lungs of COPD and IPF Patients

**Figure 4:**
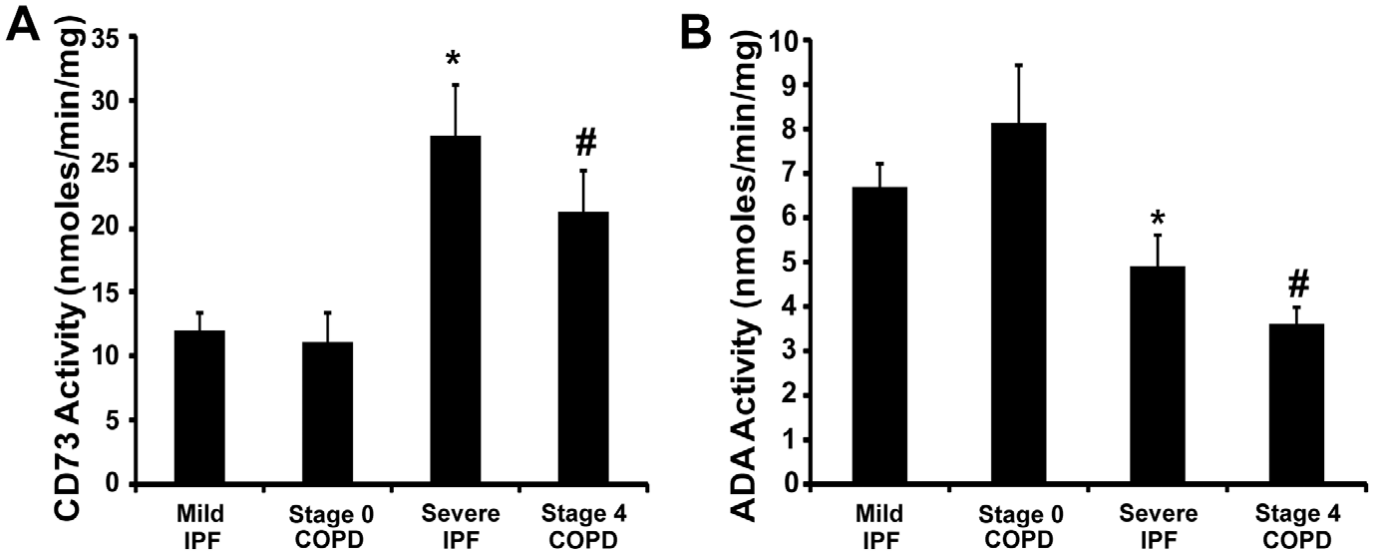
CD73 and ADA enzymatic activity. CD73 (A) and ADA (B) enzyme activity were quantified in lung protein extracts from patients. Reaction mixtures were separated, identified, and quantified by HPLC. Data are presented as mean nanomoles of substrate converted to product per min per milligram of protein ± SEM. *p≤0.05 versus Stage 0 COPD. #p≤0.05 versus Mild IPF. n = 4 (Stage 0 COPD), n = 10 (Mild IPF), n = 8 (Stage 4 COPD and Severe IPF).

To examine alterations in enzymatic activities of the key enzymes of adenosine metabolism, protein extracts were made from surgical lung biopsy specimens and enzymatic activities of CD73 and ADA were determined using HPLC. The enzymatic activity of CD73 was increased by 2 fold and 2.5 fold, respectively, in Stage 4 COPD and Severe IPF patients compared to subjects with preserved lung function (Figure 4A). This finding was consistent with the increases in CD73 transcripts noted and suggests an increased capacity for extracellular adenosine generation in Stage 4 COPD and Severe IPF patients. In addition, there was a significant decrease in ADA enzymatic activity in Severe IPF patients, and Stage 4 COPD patients (Figure 4B). This observation suggests that there may be post-transcriptional regulation of ADA activity that may contribute to permissive environment for adenosine accumulation.

### Cellular Localization of CD73 and A_2B_R Expression in Lung Tissue from COPD and IPF Patients

**Figure 5:**
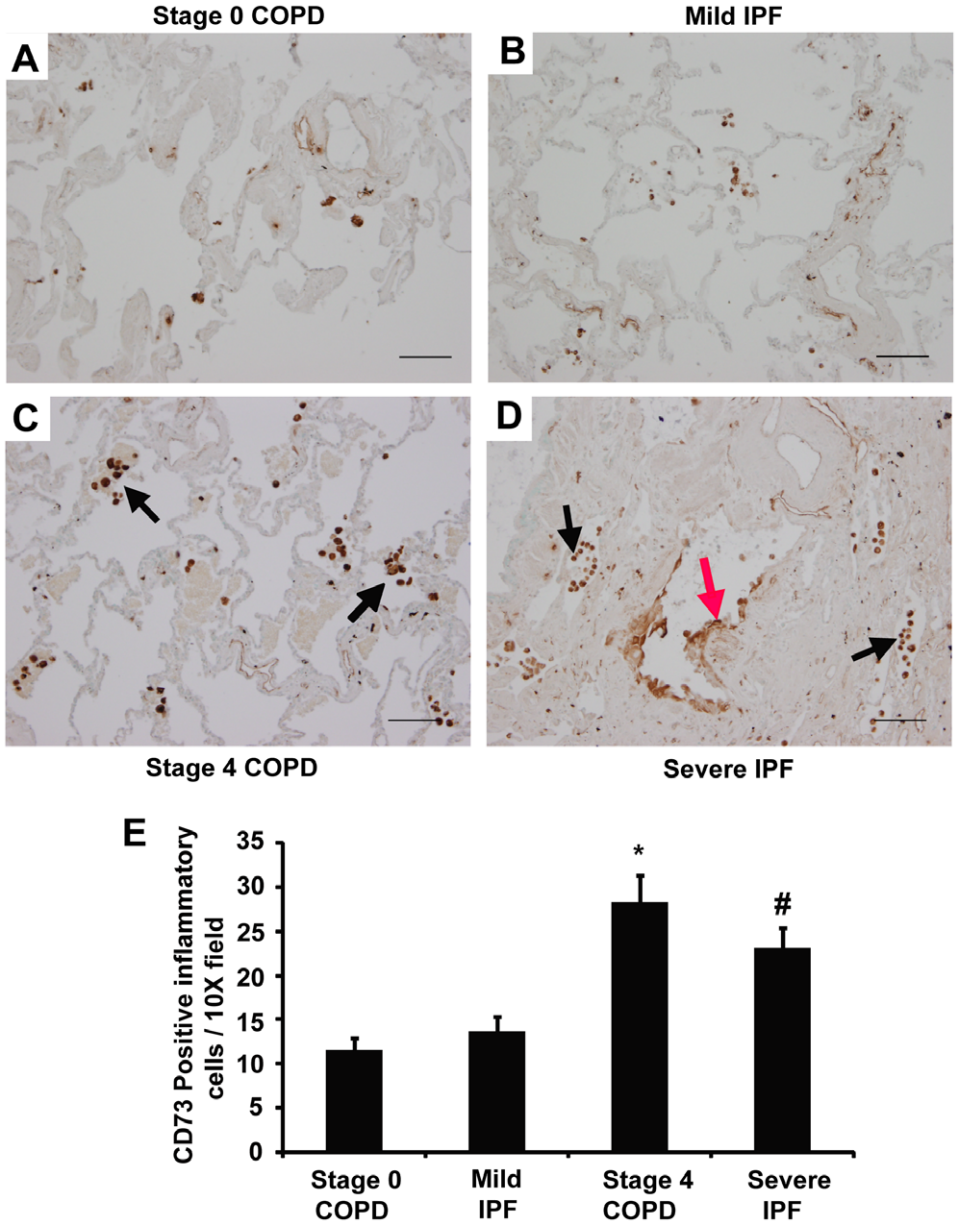
Localization of CD73. Lung sections were stained with antibodies against CD73. (A) Lung section from a Stage 0 COPD patient. (B) Lung section from a Mild IPF patient. (C) Lung section from a Stage 4 COPD patient. (D) Lung section from a Severe IPF patient. Sections are representative of 10–14 different patients from each group. Scale bars = 100 µm. (E) CD73 positive inflammatory cells were quantified in 20 images. Data are presented as mean number of positive cells per 10X field ± SEM. *p≤0.05 versus Stage 0 COPD. #p≤0.05 versus Mild IPF. n = 4 (Stage 0 COPD), n = 10 (Mild IPF), n = 8 (Stage 4 COPD and Severe IPF).

**Figure 6:**
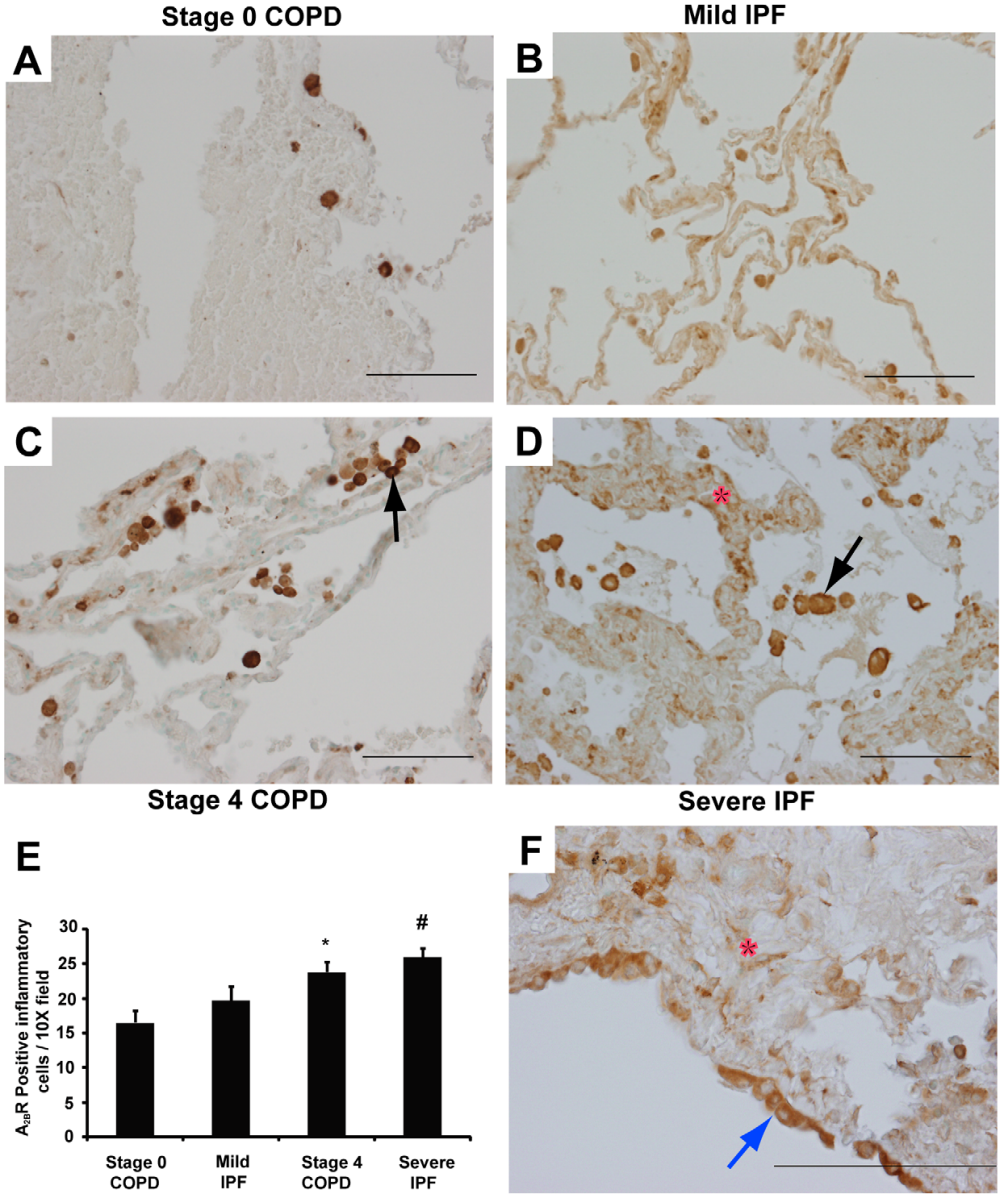
Localization of the A_2B_R. Lung sections were stained with antibodies against the A_2B_R. (A) Lung section from a Stage 0 COPD patient. (B) Lung section from a Mild IPF patient. (C) Lung section from a Stage 4 COPD patient. (D) Lung section from a Severe IPF patient. Sections are representative of 10–14 different patients from each group. Scale bars = 100 µm. (E) A_2B_R positive inflammatory cells were quantified in 20 images. Data are presented as mean number of positive cells per 10X field ± SEM. *p≤0.05 versus Stage 0 COPD. #p≤0.05 versus Mild IPF. n = 4 (Stage 0 COPD), n = 10 (Mild IPF), n = 8 (Stage 4 COPD and Severe IPF). (F) A_2B_R expression in hyperplastic airway epithelial cells (blue arrow) and fibroblasts (red asterix). Scale bar = 200 µm.

To identify the cellular localization of key components of adenosine metabolism and signaling, tissue sections from surgical lung biopsies were subjected to immunostaining with antibodies against CD73 and the A_2B_R. In subjects with preserved lung function, CD73 was expressed on inflammatory cells as well as endothelial cells (Figure 5A and 5B). In Stage 4 COPD patients, CD73 was expressed on inflammatory cells and endothelial cells (Figure 5C, arrow), while in Severe IPF patients, CD73 was localized to inflammatory cells, endothelial cells and hyperplastic cells in remodeled airways (Figure 5D, red arrow). Quantification of CD73 positive inflammatory cells revealed significant increases in Stage 4 COPD and Severe IPF patients compared to COPD and IPF patients with preserved lung function (Figure 5E). A_2B_R expression was localized to inflammatory cells, mostly macrophages, in both Stage 0 and 4 COPD patients (Figure 6A and 6C). In IPF patients, A_2B_R immunoreactivity was found on inflammatory cells, stromal cells (Figure 6B and 6D) as well as hyperplastic cells in remodeled airways (Figure 6F). Quantification of A_2B_R positive inflammatory cells revealed significant increases in Stage 4 COPD and Severe IPF patients compared to COPD and IPF patients with preserved lung function (Figure 6E).

**Figure 7:**
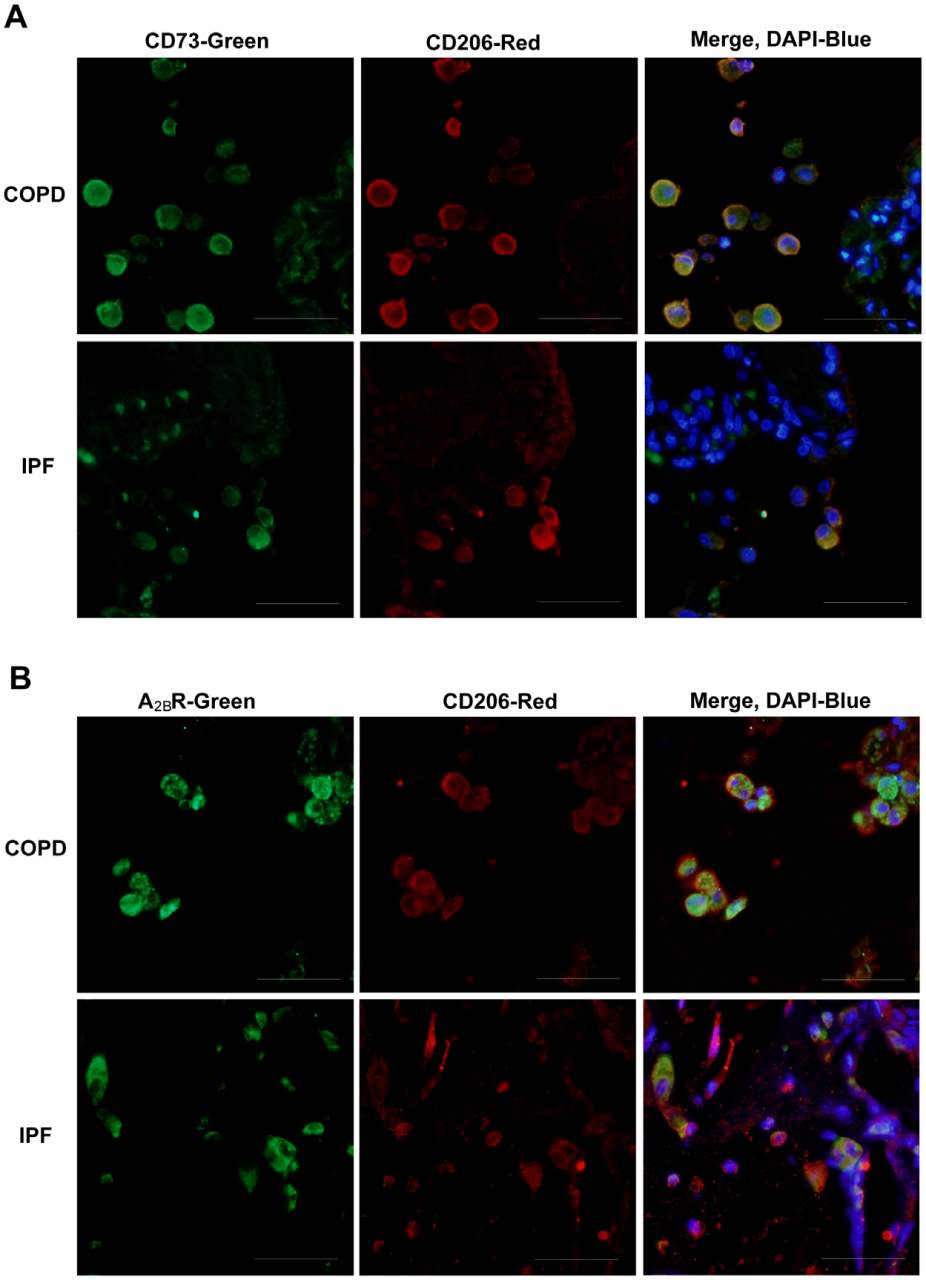
Expression of CD73 and the A_2B_R in M2 macrophages. Lung sections from COPD or IPF patients were reacted with antibodies against CD73 (A, green) or the A_2B_R (B, green) together with the M2 macrophage marker CD206 (red). In the merged images, yellow represents co-localization of CD73 or the A_2B_R and the M2 marker, blue is dapi stained nuclei. Sections are representative of 10–14 different patients from each group. Scale bars = 100 µm.

Alternatively activated macrophages, also known as M2 macrophages, are involved in microenvironments exhibiting prolonged inflammation and fibrosis, where they produce mediators that contribute to disease maintenance and progression [32], [33]. To determine if M2 macrophages within the lungs of COPD and IPF patients express CD73 and the A_2B_R, co-localization studies using an M2 macrophage marker, CD206 and either CD73 or A_2B_R antibodies were conducted on sections from these patients. Results demonstrated that M2 macrophages express both CD73 (Figure 7A) and the A_2B_R (Figure 7B) in COPD and IPF patients.

### Transcript Levels of Pro-inflammatory Mediators Downstream of Adenosine Signaling Are Altered in the Lungs of COPD and IPF Patients

**Figure 8:**
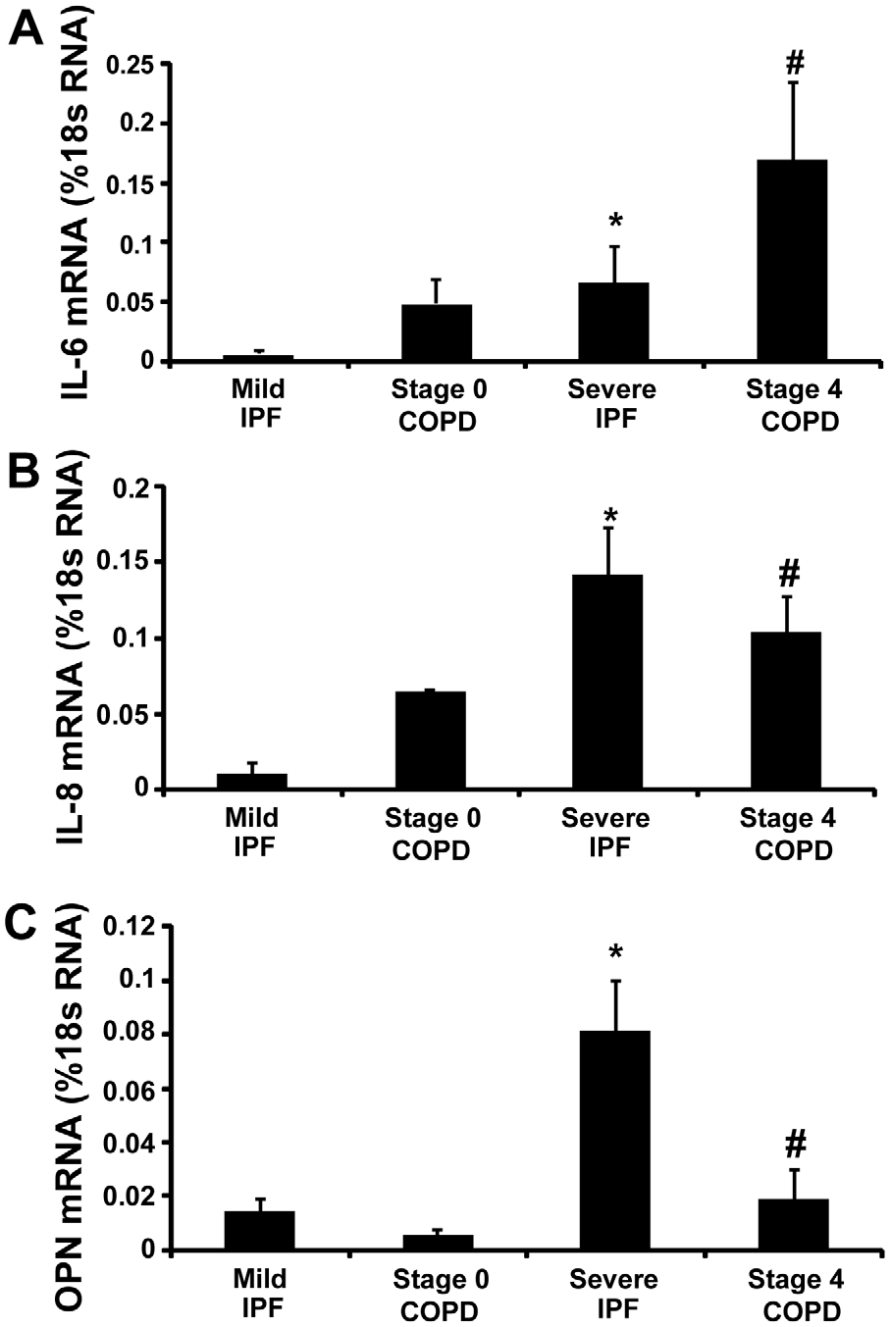
Expression of pro-inflammatory mediators. Transcript levels of various cytokines and chemokines were quantified in lung RNA extracts from patients using quantitative RT-PCR. Shown are levels of (A) IL-6, (B) IL-8, (C) OPN. Results are presented as mean percentage of 18sRNA transcripts ± SEM. *p≤0.05 versus Stage 0 COPD. #p≤0.05 versus Mild IPF. n = 4 (Stage 0 COPD), n = 10 (Mild IPF), n = 8 (Stage 4 COPD and Severe IPF).

A_2B_R signaling can regulate the expression of inflammatory and fibrotic mediators in cell types and lung tissue associated with chronic lung disease [30], [34], [35], [36], [37], [38], [39]. Total RNA was isolated from lung biopsy specimens and real time RT-PCR was performed to quantify key mediators that have been shown to be regulated by A_2B_R signaling. Results demonstrated that transcript levels of IL-6, IL-8 and OPN were all increased in both Stage 4 COPD and Severe IPF patients compared to subjects with preserved lung function (Figure 8A-C). These findings demonstrate that pro-inflammatory and pro-fibrotic mediators that can be regulated by A_2B_R signaling are elevated in COPD and IPF patients that exhibit increased A_2B_R expression.

**Figure 9:**
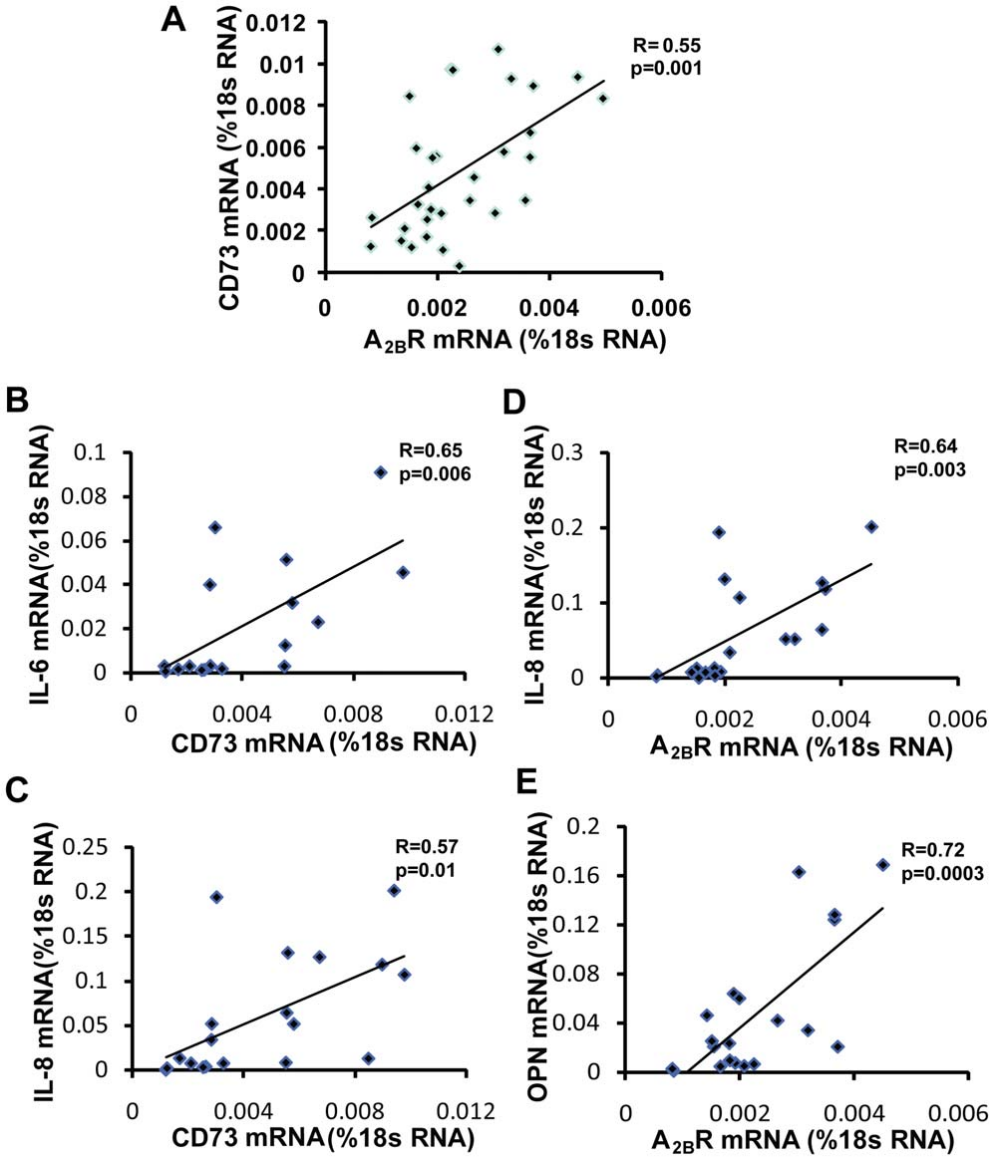
Associations between the expression of CD73, A_2B_R and inflammatory mediators. The transcript levels of CD73 in individual COPD and IPF patients were significantly associated with transcript levels of the A_2B_R (A). In IPF patients, the transcript levels of IL-6 (B) and IL-8 (C) were significantly correlated with the transcript levels of CD73; the transcript levels of IL-8 (D) and OPN (E) were significantly correlated with the transcript levels of A_2B_R.

To investigate the associations between these components of adenosine metabolism and signaling and downstream effecter molecules, correlations between A_2B_R, CD73 and inflammatory/fibrotic mediator transcript levels were determined using linear regression. CD73 levels demonstrated a significant correlation with A_2B_R transcript levels (Figure 9A). Although significant correlations were not found in COPD patients, in IPF patients, the transcript levels of IL-6 and IL-8 exhibited a significant correlation with CD73 (Figure 9B and C), while the transcript levels of IL-8 and OPN significantly correlated with A_2B_R expression (Figure 9D and E).

### A_2B_R Signaling on M2 Macrophages Contributes to the Production of IL-8 and IL-6

**Figure 10:**
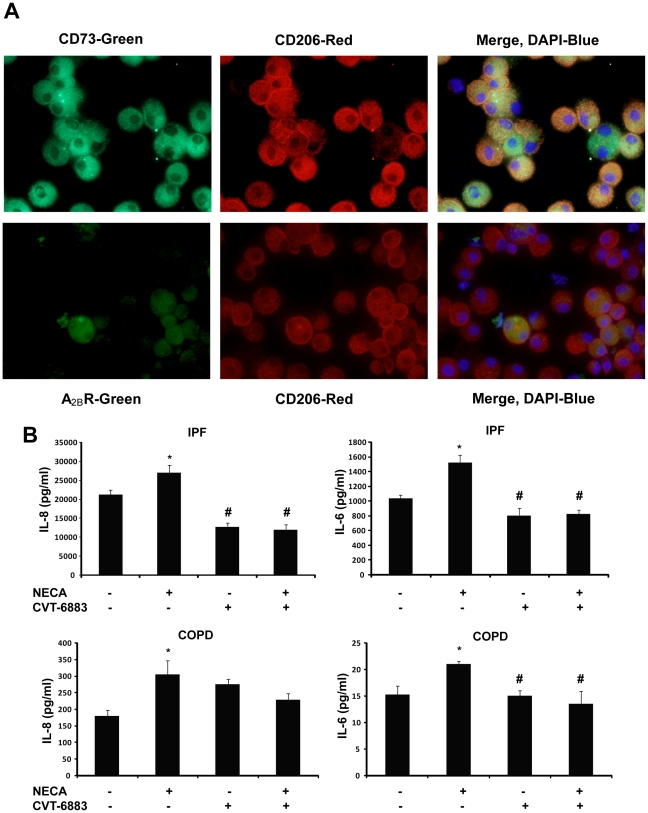
A_2B_R-dependent IL-8 and IL-6 expression in human primary alveolar macrophages. (A) Cells from an IPF patient were reacted with antibodies against CD73 (A, *upper panel*, green) or the A_2B_R (A, *lower panel*, green) together with the M2 macrophage marker CD206 (red). In the merged images, yellow represents co-localization of CD73 or the A_2B_R and the M2 marker, blue is dapi stained nuclei. (B) ELISA measurements of IL-8 and IL-6 production from macrophage cultures of IPF and COPD patients. Results are presented as mean concentrations of cytokines ± SEM. *p≤0.05 versus cells without any treatment. ^#^p≤0.05 versus cells treated with NECA alone. n = 6.

To determine if the A_2B_R on M2 macrophages is directly involved in the induction of pro-inflammatory/fibrotic mediators, human primary alveolar macrophages were isolated from the BAL fluid of Severe IPF and Stage 4 COPD patients. Co-localization studies using an M2 macrophage marker, CD206 and either CD73 or A_2B_R antibodies were conducted on cells isolated from a patient with IPF to validate that the isolated cells were predominantly M2 macrophages and to demonstrate that they express both CD73 and the A_2B_R (Figure 10A). Primary alveolar macrophages were placed in culture and pretreated with the A_2B_R antagonist (CVT-6883) before NECA exposure. This resulted in a loss of NECA-stimulated increases in IL-8 and IL-6 production (Figure 10B). Interestingly, A_2B_R antagonism alone was able to decrease baseline production of IL-8 and IL-6 in cell cultures from the IPF patients, suggesting these cells are already activated and adenosine produced from these cells can activate the A_2B_R and contribute to IL-8 and IL-6 production (Figure 10B). These findings demonstrate that IL-8 and IL-6 expression is regulated by activation of the A_2B_R on M2 alveolar macrophages in the lungs of IPF and COPD patients.

## Discussion

Adenosine is a signaling molecule produced as a result of cell stress or damage. Several studies demonstrate elevated adenosine levels in patients with chronic lung disease. Adenosine levels are elevated in lavage fluid collected from asthmatics [4], in the exhaled breath condensate of patients with allergic asthma [5], in plasma of asthmatic subjects following bronchial provocation with allergen [40], and in patients with exercise-induced asthma [41]. In addition to asthma, adenosine levels are elevated in sputum samples from cystic fibrosis patients [42]. Consistent with this, adenosine levels are elevated in various mouse models exhibiting features of chronic lung disease [23], [24], [25], [26]. In addition, the levels of key components of adenosine metabolism and signaling are altered in these mouse models in a manner that promotes adenosine accumulation and tissue-destructive adenosine receptor signaling, a process known as “purinergic remodeling”. Our hypothesis was that purinergic remodeling also exists in patients with Stage 4 COPD and/or Severe IPF relative to patients with less severe forms of these diseases. Confirmation of this would provide proof of concept information that chronic lung diseases such as COPD and IPF may benefit from treatment with adenosine based therapeutics.

CD73 is the major enzyme for extracellular adenosine production. CD73 levels are up-regulated in the lungs of mouse models with chronic lung disease including ADA-deficient mice and mice exposed to bleomycin [25], [27]. In addition, bronchial epithelial cells from patients with cystic fibrosis exhibit increased CD73 activity [43]. These findings suggest that the up-regulation of CD73 is an important purinergic remodeling response in environments where adenosine has been shown to regulate disease. In the current study, we observed that transcript levels of CD73 are elevated in both Stage 4 COPD and Severe IPF patients compared with subjects with preserved lung function. Consistent with this, the enzymatic activity of CD73 was significantly increased in both Stage 4 COPD and Severe IPF patients. CD73 was expressed on endothelial cells and inflammatory cells in normal lungs, and endothelial cells, inflammatory cells and hyperplastic epithelial cells in remodeled airways in diseased lungs. These findings suggest that there is an increased capacity for adenosine production in the lungs of Stage 4 COPD and Severe IPF patients. The levels of adenosine have not been measured in the lungs of COPD and IPF patients due to problems associated with the rapid metabolism of adenine nucleotides and adenosine. The ability to measure increases in CD73 provides a novel and important means of determining the capacity of adenosine generation. This study is the first to demonstrate this in the lungs of patients with Stage 4 COPD and Severe IPF and suggest these diseases are associated with increases in adenosine levels.

ADA is the major enzyme of adenosine metabolism [44]. It deaminates adenosine to inosine both intracellularly and extracellularly. Mice over-expressing the Th2 cytokines IL-4 or IL-13 in their lungs exhibit features of COPD and pulmonary fibrosis in association with adenosine elevations [23], [24]. Interestingly, ADA transcripts and enzymatic activity are selectively down-regulated in the lungs of these mice suggesting a purinergic remodeling response directed at promoting adenosine accumulations. Our findings demonstrate that although the transcript levels of ADA are not decreased in Severe IPF and Stage 4 COPD patients, the enzymatic activity of ADA is significantly reduced in these patients. ADA has been shown to be regulated both transcirptionally and post-transcriptionally [45]. Our results suggest that post-transcriptional regulation of ADA mRNA might contribute to the regulation of ADA in the lungs of IPF and COPD patients. Potential mechanisms of post-transcriptional regulation include the regulation of ADA mRNA translation efficiency by microRNAs, enhanced protein stability or covalent modifications to the protein such as phosphorylation, that could increase its enzymatic activity. Little is known about the role of these processes in regulating ADA enzymatic activity, and examining these mechanisms could provide important insight into novel mechanisms of purinergic remodeling in disease. Collectively, our findings suggest that there is less adenosine metabolism in the lungs of Severe IPF and Stage 4 COPD patients, which could contribute to adenosine accumulations.

Adenosine regulates numerous cellular activities by engaging cell surface adenosine receptors [8]. Research conducted by us and others have provided evidence that adenosine receptor expression is altered in models of lung disease. The A_1_R, A_2B_R and A_3_R are elevated in the lungs of models exhibiting pulmonary fibrosis and alveolar airspace destruction [23], [24], [29], [46]. Interestingly, the A_2A_R, which is down-regulated in these models, is largely an anti-inflammatory adenosine receptor [47]. The differential expression of these receptors in models of lung disease provides insight into the potential role of adenosine signaling in COPD and pulmonary fibrosis. In the current study, transcript levels of the A_2B_R were significantly elevated in both Stage 4 COPD and Severe IPF patients. A_2B_R expression was localized predominantly to inflammatory cells with some expression noted on remodeled airway epithelial cells and fibroblasts in IPF patients. The findings in IPF patients were consistent with a recent study demonstrating that A_2B_R levels are elevated in a subset of patients with rapidly progressing IPF [12]. In addition, our findings demonstrating immunoreactivity in macrophages found in the airways of COPD patients are consistent with a recent study by Varani and colleagues [11]. However, there were differences between the two studies regarding the relative levels of A_2B_R transcripts. Whereas we demonstrate increases in only the A_2B_R in the lungs of Stage 4 COPD patients, Varani and colleagues demonstrated increases in transcript levels for the A_2A_R and A_3_R and decreased levels of the A_2B_R [11]. The reason for these discrepancies are not clear, but may be related to differences in disease severity and/or smoking status of the patients in these two studies.

The A_2B_R has the lowest affinity for adenosine and is therefore likely activated under pathological conditions where adenosine levels are increased [48]. A_2B_R activation has both anti- and pro-inflammatory actions and both anti- and pro-fibrotic roles [7], [49]. Despite the receptor's homeostatic and protective functions during the acute phase of lung injury [38], [50], several studies have shown that signaling through the A_2B_R can contribute to the pathology and progressive nature of chronic lung diseases [20], [30], [39]. For example, activation of the A_2B_R on pulmonary fibroblasts promotes their differentiation into myofibroblasts and hence increases the deposition of collagen and fibrosis [20]. Furthermore, A_2B_R signaling increases the expression of fibronectin in airway epithelial cells [22]. Activation of the A_2B_R can also promote the expression or release of pro-inflammatory cytokines such as IL-4, IL-8 and IL-13 [34], [35], [36], which may influence the chronic nature of certain lung diseases. Moreover, the A_2B_R is responsible for promoting the expression of IL-6, OPN and CXCL1 (mouse homologue of IL-8) in mouse alveolar macrophages in lungs exhibiting airspace enlargement and fibrosis [30], [51]. Consistent with these findings, we demonstrate here that transcript levels of IL-6, OPN and IL-8 are elevated in the lungs of Stage 4 COPD and Severe IPF patients. Moreover, we demonstrate that treatment with a selective A_2B_R antagonist, CVT-6883 can prevent the release of IL-6 and IL-8 from M2 macrophages isolated from these patients. These findings, together with published observations that CVT-6883 can reduce the levels of these mediators and prevent fibrosis and airspace enlargement in mouse models of pulmonary injury [30], suggest that this antagonist may be useful in treating patients with COPD and IPF.

Although evidence of purinergic remodeling and A_2B_R driven mediator expression was found in both IPF and COPD patients, it is important to note that there were differences in these features between diseases. For example, association studies demonstrated a close relationship between CD73 or A_2B_R expression and IL-6, IL-8 and OPN expression in IPF patients but not in COPD patients. This suggests that although purinergic remodeling exists in both COPD and IPF patients, it is more likely that there is a causative role for A_2B_R driven mediator expression in IPF than in COPD. Alternatively, CD73 and A_2B_R differences might correlate with yet uncharacterized responses that are more closely associated with COPD, such as protease production. Thus, purinergic remodeling responses in IPF and COPD exists to increase adenosine levels; however, the downstream consequences likely differ amongst disorders with different pathological manifestations.

Substantial evidence in mouse models of chronic lung disease suggests that adenosine-based therapeutics are beneficial for the treatment of chronic lung disease where airspace destruction and fibrosis is prominent [7]. ADA replacement therapy consists of intra-peritoneal or intranasal treatment with ADA conjugated to polyethylene glycol. This therapy is able to prevent elevations in adenosine, or lower the levels of elevated adenosine in various models of lung disease [23], [24], [27], [29]. Preventing elevations in adenosine using this therapy can prevent and reverse airway inflammation, fibrosis, and alveolar airspace enlargement. Thus, adenosine elevations play an important role in accessing pathways that lead to pulmonary fibrosis and airspace destruction. The ADA-deficient model of adenosine-dependent pulmonary disease has provided a useful mechanism for examining the contribution of individual adenosine receptors to the inflammation and damage seen in the lung in response to adenosine elevations. A recent study used a selective A_2B_R antagonist (CVT-6883) to block this receptor in ADA-deficient mice [30]. Treating ADA-deficient mice with this antagonist prevented the production of numerous mediators from alveolar macrophages, thus preventing the development of airspace enlargement and pulmonary fibrosis [30]. Treatment with CVT-6883 also prevented pulmonary fibrosis in the bleomycin induced fibrosis model [30]. These findings demonstrate that elevations in adenosine in the lung can promote airspace enlargement and fibrosis in part by engaging the A_2B_R. These findings in mouse models raised the possibility that ADA replacement therapy and A_2B_R antagonist treatment may benefit patients with airspace enlargement and pulmonary fibrosis.

In conclusion, findings in the current study suggest that components of adenosine metabolism and signaling are altered in a manner that promotes adenosine production in patients with Stage 4 COPD and Severe IPF. These changes include the up-regulation of CD73, a down-regulation of ADA activity, and elevations in the A_2B_R. It provides proof of concept information that human COPD and IPF patients may benefit from adenosine-based therapeutics such as ADA enzyme replacement therapy or treatment with an A_2B_R antagonist. In addition, monitoring purinergic remodeling responses in lung samples provides an attractive approach for screening patients for the potential effectiveness of adenosine-based therapeutics.
